# Parametric scheme for rapid nanopattern replication *via* electrohydrodynamic instability†

**DOI:** 10.1039/d1ra01728d

**Published:** 2021-05-19

**Authors:** Jaeseok Hwang, Hyunje Park, Jaejong Lee, Dae Joon Kang

**Affiliations:** Department of Energy Science, Sungkyunkwan University 2066, Seobu-ro, Jangan-gu Suwon Gyeonggi-do 16419 Republic of Korea; Department of Physics, Sungkyunkwan University 2066, Seobu-ro, Jangan-gu Suwon Gyeonggi-do 16419 Republic of Korea djkang@skku.edu; Korea Institute of Machinery and Materials (KIMM) 156 Gajeongbuk-ro, Yuseong-gu Daejeon 34103 Republic of Korea jjlee@kimm.re.kr

## Abstract

Electrohydrodynamic (EHD) instability patterning exhibits substantial potential for application as a next-generation lithographic technique; nevertheless, its development continues to be hindered by the lack of process parameter controllability, especially when replicating sub-microscale pattern features. In this paper, a new parametric guide is introduced. It features an expanded range of valid parameters by increasing the pattern growth velocity, thereby facilitating reproducible EHD-driven patterning for perfect nanopattern replication. Compared with conventional EHD-driven patterning, the rapid patterning approach not only shortens the patterning time but also exhibits enhanced scalability for replicating small and geometrically diverse features. Numerical analyses and simulations are performed to elucidate the interplay between the pattern growth velocity, fidelity of the replicated features, and boundary between the domains of suitable and unsuitable parametric conditions in EHD-driven patterning. The developed rapid route facilitates nanopattern replication using EHD instability with a wide range of suitable parameters and further opens up many opportunities for device applications using tailor-made nanostructures in an effective and straightforward manner.

## Abstract 2

1/*τ*_m_-dependent electrohydrodynamic replication of a hexagonally ordered hole array nanopattern by adjusting the filling ratio. As the 1/*τ*_m_ increases, the morphology evolves into the perfectly replicated hole features with increasing filling ratio.

## Graphical abstract

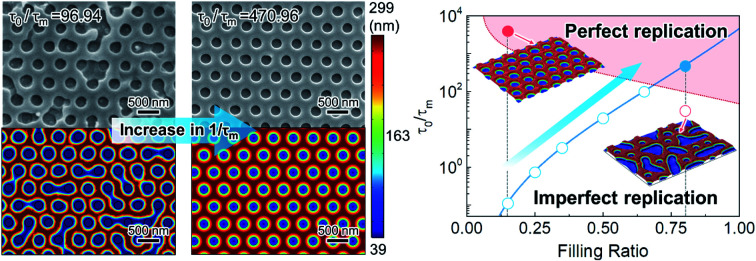

## Introduction

Various fabrication techniques have been extensively studied for decades,^1–6^ aimed at producing finely structured surfaces for a wide range of applications, such as memory devices,^6,7^ micro-/nanofluidic devices,^8,9^ optical devices,^10^ sensors,^11,12^ and diverse functional coatings.^13,14^ Among them, instability of fluidic thin film can be regarded as a viable alternative to conventional top-down approaches, due to scalability, cost-effectiveness, and versatility.^15^ As one example, without any expensive equipment or meticulous procedure, surface instability engendered by van der Waals interactions leads to the morphological evolution from an initially flat feature to an array of holes or droplet shape to reduce the excessive free energy of the system.^16^ However, this fabrication technique using the spontaneous instability is limited to only the very thin film (a few tens of nanometer thickness) and poses a great challenge to achieving geometrically diverse and regular nanopatterns.^15^

With regard to overcoming the limitations of spontaneous instability mediated by interfacial disjoining pressure and high cost of state-of-the-art lithographic techniques, electrohydrodynamic (EHD)-driven patterning, a novel bottom-up pattern fabrication using the electric field-mediated instability, has drawn substantial attention ever since the pioneering work by Steiner's group.^17^ EHD instability (*i.e.*, electric field-mediated instability) is not merely exploited for pattern transfer in a single-step, non-contact, versatile, and scalable manner,^18–24^ but also provide diverse opportunities for finely tailoring the structural property by carefully selecting process parameters.^25–31^

EHD-driven patterning is based on the phase instability behavior of a thin liquid film under an applied out-of-plane electric field that induces undulation of the liquid film surface to form small patterns; this has been demonstrated experimentally *via* the fabrication of micro and nanoscale patterns.^17,32,33^ In the EHD-driven patterning, electrostatic pressure acting on the surface of the fluidic film plays a critical role in film destabilization. However, while the film surface deforms to reduce the electrostatic energy stored in capacitor assembly, the Laplace pressure caused by the surface curvature is generated to stabilize the undulating film surface. This interplay has traditionally been characterized by the critical wavelength (*λ*_m_), which is defined as the wavelength of the fastest-growing mode in the surface undulation; particularly, *λ*_m_ is related with the forces acting on the liquid thin film–air interface, and it is estimated as follows:^20^1
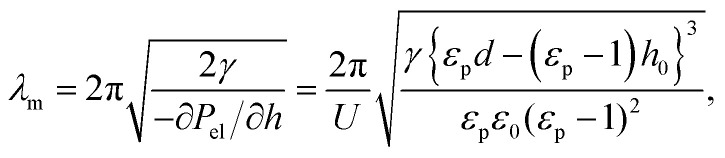
where *U*, *d*, *h*_0_, *γ*, *ε*_0_, *ε*_p_, and *P*_el_ are the applied voltage, electrode spacing, initial film thickness, surface tension coefficient, vacuum permittivity, dielectric constant, and electrostatic pressure 
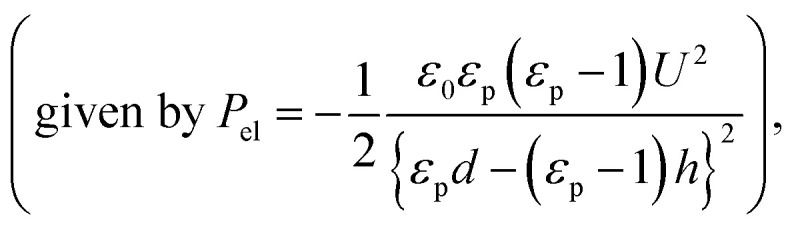
 respectively. These parameters must be carefully chosen because conventionally, the resulting *λ*_m_ critically affects the final morphology and temporal evolution of the surface undulations.

The underlying mechanism for pattern replication induced by EHD instability in the fluidic thin film has been extensively studied.^34–37^ When using EHD-driven patterning to replicate a pattern, a topographically structured top electrode (master stamp) is used (instead of a planar featureless top electrode), which results in the generation of a laterally modulated electric field that locally enhances the instability of the liquid thin film, thereby guiding positive replicated pattern formation in the liquid thin film.^17^ A parametric guide for perfect replication was first proposed based on the influence of the master pattern periodicity *λ*_p_ on the pattern replication;^38,39^*λ*_m_ must be adjusted such that it is on a scale similar to that of *λ*_p_, which is known as *λ*_m_–*λ*_p_ matching. Generally, *λ*_m_–*λ*_p_ matching poses a significant challenge to facile and reliable nanoscale EHD-driven patterning because it is relatively difficult to precisely match *λ*_m_ with nanoscale *λ*_p_ owing to the limited range of process parameters for *λ*_m_. For instance, although increasing the applied voltage (*U*) is favorable for reducing *λ*_m_, an excessively high voltage causes electric breakdown across the nanoscale electrode separation (*d*).

In their work using low-viscosity polymers, Goldberg-Oppenheimer *et al.*^40^ demonstrated that the influence of *λ*_m_ on successful replication is seemingly negligible during rapid patterning (<10 s) without compromising the fidelity of the replicated micropatterns, and they highlighted the potential for nanoscale patterning *via* accelerated EHD-driven patterning. However, their approach is limited to low-viscosity materials, and they did not present rapid EHD-driven patterning as a general strategy for nanoscale and microscale patterning in a facile and controllable manner. Further, the validity of their parameter scheme for highly reproducible EHD-driven patterning and the conditions under which dense nanoscale patterns can be replicated through an accelerated rapid approach remain unexplored.

Nonetheless, their results suggest that the speed of the pattern evolution may be more critical to the pattern growth than *λ*_m_ and that more facile and effective EHD-driven patterning might be achieved by reducing the processing time, *i.e.*, accelerating the spatiotemporal evolution of the pattern. Focusing on this, we hypothesize that at the initial stage of the pattern growth, amplifying the surface undulation enables successful replication of nanopatterns regardless of *λ*_m_–*λ*_p_ mismatch. To verify this, our study aimed to elucidate the manner in which the fidelity of the replicated nanopatterns is influenced by the reciprocal of the characteristic time parameter (1/*τ*_m_); to this end, certain aspects of the patterning process, such as temperature, ratio of initial film thickness to electrode distance, and master pattern feature height (*h*_p_), were systematically adjusted. Based on our experimental results and simulation-based analysis, we found that parameter control in EHD-driven patterning can be significantly improved and facilitated by increasing 1/*τ*_m_. The corresponding rapid patterning approach facilitates nanopattern replication with a broader range of process parameters than what is possible in conventional EHD-driven patterning. Accordingly, we demarcate the parameter ranges corresponding to perfect and imperfect replication regimes with respect to 1/*τ*_m_. By studying 1/*τ*_m_, important rheological information regarding the destabilized liquid thin film is obtained. This result serves as a technical guide for achieving highly reproducible replication of small patterns down to the sub-10 nm level and explains the significant advantage of parameter control in EHD-driven patterning in detail.

## Experimental

### EHD patterning process

All chemicals were purchased from Sigma-Aldrich. For the parameter control experiments, PS (average *M*_W_ = 192 000) was used as a proof-of-concept film material for EHD-driven patterning in all cases to minimize the effect of a high dielectric constant (*ε*_PS_ = 2.5). PS was dissolved in toluene (concentration 1.5–5 wt%). The PS solution was filtered through a 0.2 μm Millipore membrane and spin cast onto a pre-cleaned silicon substrate. Highly p-doped silicon wafers were employed as conductive substrates (bottom electrode area 2 × 2 cm^2^). Prior to spin coating, the substrates were cleaned in a Piranha solution with a 3 : 1 ratio of H_2_SO_4_ (98%) and H_2_O_2_ (30%) at 150 °C for 30 min, followed by rinsing in deionized water. Here, a 200 nm-thick PS thin film was used to maintain *f* = 0.5 (*d* was fixed at 400 nm). However, in the filling ratio control experiment, different PS film thicknesses (60 to 360 nm) were employed by varying the concentration and spin coating rpm.

Various types of master patterns (2 × 2 cm^2^) were prepared for each experiment. To investigate the temperature control, highly ordered line arrays with a 300 nm linewidth and 600 nm periodicity and hexagonally ordered pillar arrays with a 150 nm diameter and 400 nm periodicity (center-to-center distance) were used as master patterns. Hexagonally ordered hole arrays with a 300 nm diameter and 500 nm periodicity were used as a master pattern in the filling ratio control experiments, and line arrays with the same linewidth (300 nm) but different heights (40, 120, or 300 nm) created *via* etching were used in the master stamp protrusion height control experiments. In the scalability control experiments, two types of master pattern shapes were selected. In the line array pattern, the linewidths were 150, 200, and 300 nm, and in the hexagonal pillar pattern, the pillar diameter and periodicity were 150 nm and 300, 400, or 700 nm, respectively. All master patterns used in this work were fabricated using electron-beam lithography and etching to transfer the pre-pattern onto silicon. To minimize the accumulation of PS residue on the master patterns in the event of PS-master protrusion contact, all master stamps were treated with a vapor-phase self-assembled monolayer of 1,1,1,2*H* perfluorodecyltrichlorosilane to enhance hydrophobicity.

A power supply capable of controlling a <5 mA current and producing a DC voltage of <200 V was used during EHD-driven patterning. As in typical processes, a PS thin film was sandwiched between the top (master stamp) and bottom electrodes, leaving an air gap of <500 nm created by SiO_2_ spacers mounted on the master stamp (see Fig. S1a†). A nanoimprinter (model ANT-2T developed by the Nano-Mechanical System Research Division of the Korea Institute of Machinery and Materials) was employed to maintain a uniform air gap over the entire PS film surface (see Fig. S1b†). The PS thin film was heated above its glass transition temperature (*ca.* 100 °C) to make it malleable and evaporate the solvent. In particular, for the temperature control experiments, the top and bottom electrodes were heated to the same temperature (120, 170, 250, and 350 °C) to avoid any effects arising from a temperature gradient normal to the film surface, as the flux of thermal energy can destabilize the film surface.^41^ A DC voltage <120 V was applied across the electrode for a processing time of 15 min. At high filling ratios (*f* > 0.65), the duration for EHD-driven patterning was limited to <5 min (at 60 V) owing to frequent current leakage across the two electrodes.

Samples fabricated by EHD-driven patterning were observed using a field emission SEM (JEOL JSM7401F) at acceleration voltages of 3.0–5.0 kV to examine the pattern morphology. Topological information was obtained using an atomic force microscope (AFM, Veeco, Dimension 3100) in the tapping mode.

Numerical analysis was performed using Mathematica ver. 11.01. The COMSOL Multiphysics 5.3 software package was used to conduct the simulations based on a partial differential equation (PDE) mode. By inserting the summation of the electrostatic pressure and van der Waals force into a source term in a PDE, two time-dependent variables, the interfacial height (*h*(*x*, *y*, *t*)) and overall pressure on the film surface (*P*(*h*, *t*)) were solved. It should be noted that the effect on the van der Waals interaction was ignored due to the film thickness (*i.e.*, 200 nm) and strong electrostatic pressure. In the simulations, and the number of nodes was approximately 10^3^, which was sufficient enough to result in < 1% relative error. Periodic boundary conditions were applied at the edges of the simulation domain such that it was spatially connected to itself, thereby eventually creating a large domain. Various physical quantities, such as film height, surface pressure, and fluid flux, were calculated simultaneously, and the results were expressed as 2D or 3D images to facilitate their comparison with the experimental results.

## Results and discussion

**Fig. 1:**
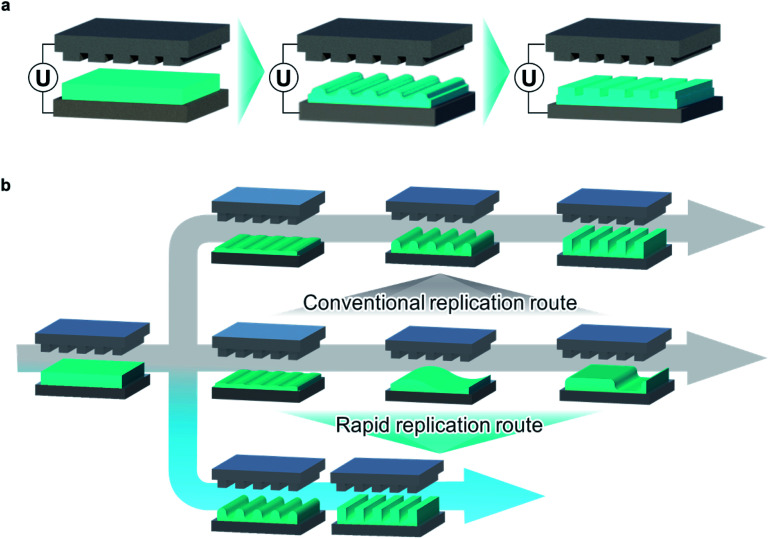
A schematic diagram of the time-dependent replication process driven by electrohydrodynamic instability with different replication routes. (a) When an electric potential *U* is applied across the patterned top electrode and the bottom substrate, the laterally modulated electric field locally induces electrohydrodynamic instability where the field strength is strongest, leading to ripples on the liquid thin film surface. Over the processing time, the ripples develop to form a pattern that is identical to the given master pattern on the top electrode. (b) To perfectly replicate the master pattern, the precise control of experimental parameters for *λ*_m_/*λ*_p_ ≈ 1 needs to be pursued in conventional replication (top row) route while avoiding *λ*_m_/*λ*_p_ ≫ 1 (middle row). When *λ*_m_ is larger than the periodicity of the master pattern *λ*_p_ (middle row), the pattern will be partially replicated or fail to replicate the master pattern. However, with a rapid replication route, increasing 1/*τ*_m_ enables perfect replication regardless of the values of *λ*_m_/*λ*_p_ (bottom row).

A typical EHD-driven patterning system is set up with a focus on precise control of the process temperature, applied voltage, liquid thin film thickness, and electrode separation; the shape and size of the master pattern also play a critical role (more details are given in the Experiments and ESI sections†). The EHD-driven patterning procedure for achieving nanostructure replication is briefly summarized as follows.^25,42,43^ First, a thin polymeric film with an initial thickness of 60–320 nm is liquefied by heating it to above its glass transition temperature, and a topologically structured top electrode (master stamp) is placed over the liquid thin film with a nanoscale air gap between them. When an electric field of >10^6^–10^7^ V m^−1^ is applied perpendicular to the thin film surface, the thin film–air interface undulation begins and is increasingly amplified over time; finally, it adopts the shape of the master stamp pattern over the processing time, as illustrated in Fig. 1a.

Successful replication is not always guaranteed, but morphologies are generally obtained depending on the ratio of the critical wavelength (*λ*_m_) to the master pattern periodicity (*λ*_p_), *λ*_m_/*λ*_p_. When *λ*_m_ is similar to the periodic interval between the master pattern features, the formed pattern is a replica of the master pattern, as shown in Fig. 1b (top row). Otherwise, if, *e.g.*, *λ*_m_ is smaller or larger than *λ*_p_, *λ*_m_ of the undulating surface has a considerable effect on the pattern replication process such that the pattern fidelity is lost (middle row), which has been observed in previous studies.^38,39^ During nanopattern replication in conventional route, *λ*_m_/*λ*_p_ > 1 is generally easier to achieve because decreasing *λ*_m_ to match or be less than nanoscale *λ*_p_ by fine-tuning the key parameters (*i.e.*, applied voltage and electrode distance) is difficult and results in unreliable nanopattern replication.

Pattern replication is not exclusively governed by *λ*_m_ throughout the entire pattern growth process. Based on simulations,^38,44^ the initial pattern formation always follows a length scale close to that of *λ*_p_ regardless of *λ*_m_/*λ*_p_ when electrohydrodynamic instability is induced by the master pattern-induced (*λ*_p_) spatially modulated electric field. However, the later stages of the time evolution are governed by *λ*_m_ as well as *λ*_p_, thus determining the final morphology of the replicated structures for long processing times. Considering the role of time in the pattern evolution, especially during the early stages, its influence should be examined in detail, with a focus on the fidelity of the replicated pattern and with respect to maintaining *λ*_m_–*λ*_p_ matching. First, we can easily notice the numerical relation between the initial velocity of pattern growth and reciprocal characteristic time 1/*τ*_m_. With the sinusoidal ansatz for the air–film interfacial height *h*, the growth velocity (∂*h*(*x*, *t*)/∂*t*) at time *t* = 0 is2
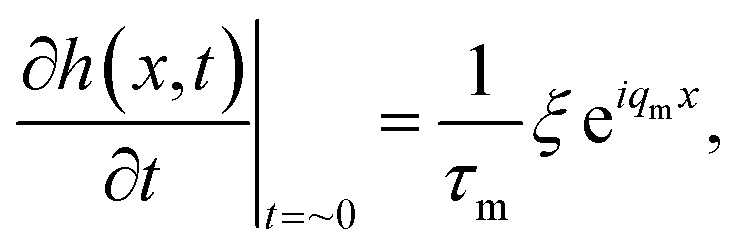
where *ξ* and *q*_m_ are the initial amplitude of the air–film interface undulation (which is negligible because *ξ* ≪ *h*_0_) and the maximum wavenumber of the fastest growing undulation (*i.e.*, critical wavelength *λ*_m_). Therefore, 1/*τ*_m_ can be considered as the initial velocity of pattern growth, 
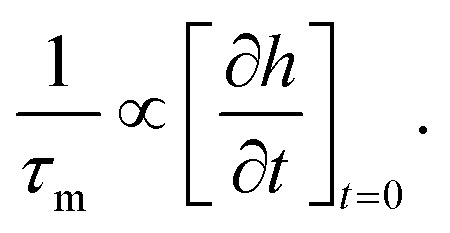


We suggest that when electrohydrodynamic instability is initiated by substantially shortening *τ*_m_ (increasing 1/*τ*_m_), *λ*_m_–*λ*_p_ matching criterion can be significantly relaxed because the initial surface undulation with features having periodicity *λ*_p_ will be rapidly amplified. In this manner, the air gap beneath the protrusions of the master stamp, which is reduced by the increased pattern height in the early stages of the time evolution of the pattern formation, further increases the electrostatic pressure, thus directing the liquid flow toward the protrusions, resulting in successful replication of the master pattern in the liquid film, as illustrated in Fig. 1b (bottom row). We derive 1/*τ*_m_ by inserting *q*_m_ into the dispersion relation:^31^3
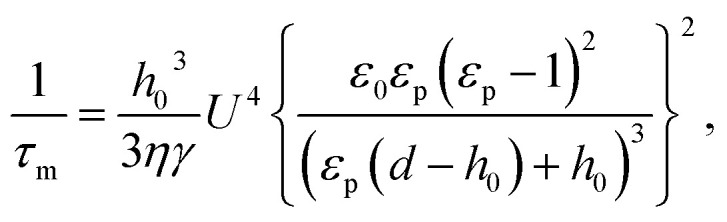
where *h*_0_, *η*, *γ*, *U*, *ε*_p_, *ε*_0_, and *d* are the initial film thickness, film viscosity, surface tension, applied voltage, dielectric constant of the film, vacuum permittivity, and distance between the two electrodes, respectively. Although it is clear that increasing *U* is effective for achieving a high 1/*τ*_m_, *U* is highly limited by electric breakdown.

Increasing the temperature (*T*) not only reduces *η* but also linearly reduces *γ* in the polymeric thin film,^45^ thereby affecting *λ*_m_ (see Fig. S2† for further details). Therefore, to investigate the influence of 1/*τ*_m_ on nanopattern replication, we first adjusted *γ* and *η* of the liquid thin film by varying *T* during EHD-driven patterning (15 min) to replicate two types of master patterns (300 nm linewidth line array with 600 nm periodicity and hexagonal array of pillars with diameters 150 nm and center-to-center distance 400 nm) on a polystyrene (PS) thin film (200 nm thick). For simplicity, 1/*τ*_0_ is defined as 1/*τ*_m_ when *λ*_m_/*λ*_p_ = 2.5 at *T* = 120 °C (*ca.* 0.253 s^−1^); *τ*_0_/*τ*_m_ is employed to express the extent to which 1/*τ*_m_ increases under a certain parametric condition.

**Fig. 2:**
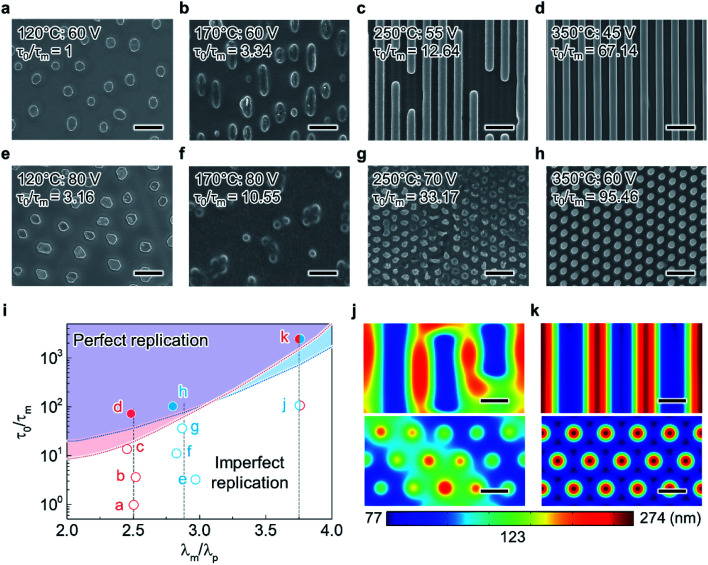
1/*τ*_m_-dependent replication of line and hexagonally ordered pillar nanostructures by varying the temperature (*T*). (a)–(h) SEM images of the replicated morphology for a range of *τ*_0_/*τ*_m_ (1–67.14 for the line pattern in (a)–(d) and 3.16–95.46 for the hexagonally ordered pillars in (e)–(h)) but similar *λ*_m_/*λ*_p_ (2.45–2.50 and 2.80–2.98, respectively), achieved by varying *T* (120 °C–350 °C) and *U*. The scale bars represent 10 μm in (a) and (e) and 1 μm in (b)–(d) and (f)–(h). (i) Relationship between *τ*_0_/*τ*_m_, *λ*_m_/*λ*_p_, and the corresponding replicated nanopattern fidelity. The filled and open circles indicate the morphologies obtained by experiment ((a)–(h)) and simulation ((j) and (k)), respectively. Red and blue circles indicate the suitable range of proper parameter conditions for obtaining perfect replication of line and hexagonally ordered pillar nanopatterns, respectively. (j) and (k) Simulated morphologies for *τ*_0_/*τ*_m_ = *ca.* 10^3^ and *ca.* 10^4^, respectively, for the same *λ*_m_/*λ*_p_ = 3.75. The scale bars represent 300 nm. The film–air interface height is indicated by the color legend.

The scanning electron microscopy (SEM) images in Fig. 2a–d and e–h show the morphology obtained *via* EHD-driven patterning as *τ*_0_/*τ*_m_ was increased (1–67.14 and 3.16–95.46, respectively) with increasing *T* (120–350 °C). At *τ*_0_/*τ*_m_ = 1 and 3.16 (*T* = 120 °C), the respective lines (Fig. 2a) and pillar structures (Fig. 2e) were not clearly observed, and there were no significant changes over the entire film surface. Nanoscale lines and pillars were imperfectly replicated at *τ*_0_/*τ*_m_ = 3.34–12.64 (Fig. 2b and c) and 10.55–33.17 (Fig. 2f and g), respectively (*T* = 170–250 °C); these conditions correspond to *λ*_m_/*λ*_p_ > 1 (*λ*_m_/*λ*_p_ = 2.45–2.5 and 2.81–2.88, respectively). As shown in Fig. 2d and h, perfect replication was obtained at *τ*_0_/*τ*_m_ = 67.14 and 95.46, respectively, resulting in replicated line and pillar patterns identical in size and shape to those of the master patterns; here, *λ*_m_/*λ*_p_ > 1 (*λ*_m_/*λ*_p_ = 2.49 and 2.80, respectively). Despite the nearly constant *λ*_m_/*λ*_p_ (*ca.* 2.5 and 2.8, respectively) in all four experiments per pattern, a slight decrease in the applied voltage together with increasing *T* enabled perfect replication of the nanopatterns.

Simulations were conducted beyond the limits of realistic EHD-driven patterning conditions; note that *γ* and *η* of the polymeric thin film are not independent of each other under different *T*. Fig. 2i shows a diagram delimiting the border between the parametric domains of suitable and unsuitable patterning conditions for each pattern. The parametric domain for imperfect replication becomes larger with increasing *λ*_m_/*λ*_p_, in accordance with the conventional approach. In contrast, the parametric domain for perfect replication (red region: line pattern; blue region: pillar pattern) becomes larger with increasing *τ*_0_/*τ*_m_. Therefore, as shown in the simulated morphology in Fig. 2j and k, nanopatterns identical in shape, size, and periodicity to those of the master patterns are perfectly replicated at *λ*_m_/*λ*_p_ = 3.75 or more when *τ*_0_/*τ*_m_ is increased by ≥ 10^3^ (see Fig. S3† for further detail).

**Fig. 3:**
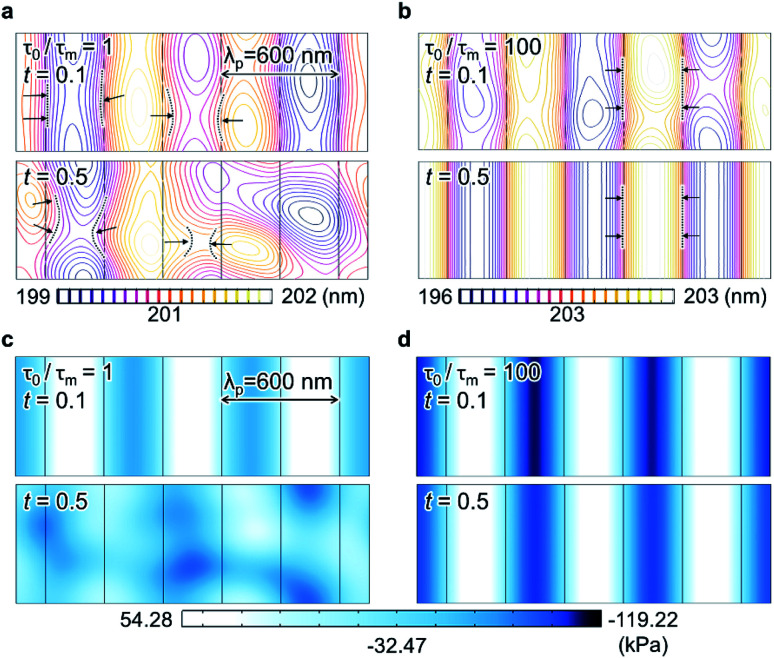
Simulation of the dielectric fluid flow for *λ*_m_–*λ*_p_ mismatching and corresponding range of *τ*_0_/*τ*_m_. (a) and (b) Subtle fluid–air interfacial variation at an early stage of the time-evolution (dimensionless time parameter *t* = 0.1) depicted by the purple-to-yellow contours and representing the periodic features (*λ*_p_) of the applied laterally varying electric field (in both cases, *λ*_m_/*λ*_p_ = 2.5). (a) At *τ*_0_/*τ*_m_ = 1, the initial interfacial variation decreased quickly (*t* = 0.5). (b) However, at *τ*_0_/*τ*_m_ = 100, the fluid was continuously transported into the pattern feature regions to form the pattern. The arrows show the direction of the fluid flow. The discrete color gradients indicate the interfacial variation. (c) and (d) Total pressure (*P*_el_ + *P*_L_) distribution across the fluid thin film (*t* = 0.1 and 0.5, respectively). For *τ*_0_/*τ*_m_ = 100 (d), the periodic feature of total pressure distribution was maintained; thus, the fluid was driven into the pattern regions. The color gradient refers to the pressure variation from 54.28 (white) to −119.22 kPa (dark blue).

To understand these results in more detail, the rheological behavior of the dielectric fluid in the electrohydrodynamic instability-induced film was simulated under a laterally varying electric field. For simplicity, all parameters, such as the applied voltage (60 V), initial film thickness (200 nm), electrode gap (400 nm), and dielectric constant (2.5), were fixed. The viscosity and surface tension were varied to increase *τ*_0_/*τ*_m_ during EHD-driven patterning. Fig. 3a and b show the initial movement of the dielectric fluid flow over dimensionless time in response to different *τ*_0_/*τ*_m_ (1 and 100, respectively), where the yellow contours indicate a higher film–air interface height and purple contours represent a lower film–air interface height. Upon inducing electrohydrodynamic instability in the fluidic thin film, the fluid was initially (*t* = 0.1, Fig. 3a and b upper images) drawn under the protrusions of the master pattern in both cases, forming lines with the same periodicity as that of the master pattern (*λ*_p_ = 600 nm). However, for *τ*_0_/*τ*_m_ = 1, which corresponds to the general case in conventional EHD-driven patterning, fluid flow was quickly directed back to the non-protrusion regions by *t* = 0.5, thus losing the initial periodicity (Fig. 3a lower image) owing to the restoring Laplace pressure. In contrast, when *τ*_0_/*τ*_m_ = 100, the fluid pattern continuously grew under the master pattern protrusions while preserving the initial periodicity to replicate the line pattern, as shown in Fig. 3b. This distinct behavior of dielectric fluids stems from the strength of the total pressure, which is defined as the sum of the destabilizing electrostatic pressure 
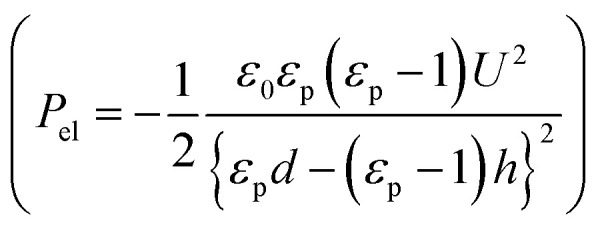
 and restoring Laplace pressure (*P*_L_ = *γ*∇^2^*h*). Increasing *T* reduces *γ* in the liquid thin film, thus decreasing *P*_L_. Moreover, it increases the initial height of the growing pattern (*h*_0_) by increasing 1/*τ*_m_ (*i.e.*, decreasing *η*); hence, the reduced air gap (*d* − *h*) further increases the strength of the electrostatic pressure, according to *P*_el_ ∝ (*d* − *h*)^−2^.

To support this argument, the spatial distribution of the total pressure was simulated during the initial stages of EHD-driven patterning, where *τ*_0_/*τ*_m_ was set to 1 and 100. For *τ*_0_/*τ*_m_ = 1, although the total pressure was periodically distributed across the spatial domain at an early stage (*t* = 0.1), it was adjusted to decrease the pressure difference, as shown in the lower image of Fig. 3c. However, when *τ*_0_/*τ*_m_ = 100, the total pressure distribution was not only periodic at an early stage but also retained the initial periodicity over the pattern growth time (Fig. 3d).

The influences of the electrode gap (*d*) and initial film thickness (*h*_0_) were investigated because both are crucial parameters in determining 1/*τ*_m_ according to eqn (3) (1/*τ*_m_ ∝ (*d* − *h*_0_)^−6^). To observe the clear impact of *d* and *h*_0_ on the pattern replication, the master pattern was chosen as a hexagonal hole array pattern with a hole diameter of 300 nm and hole-to-hole spacing of 500 nm. Note that for this master stamp, the protrusions are continuously connected around the holes, making the periodicity of the strongest field under the protrusions (*λ*_p_) meaningless. The filling ratio (*f* = *h*_0_/*d*) was adjusted from 0.15 to 0.8, and *τ*_0_/*τ*_m_ ranged from 0.11 to 470.96. For high *f* (>0.65), the EHD-driven patterning time was strictly limited to <5 min at 60 V because of the frequent leakage current occurring across the two electrodes.

**Fig. 4:**
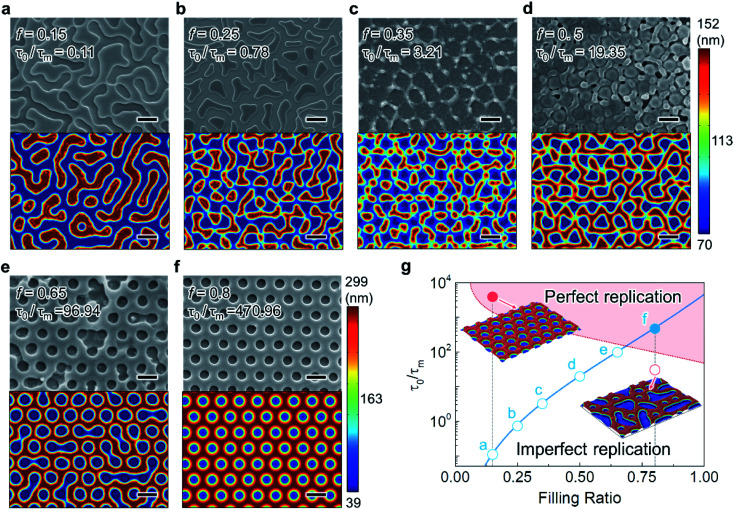
1/*τ*_m_-dependent electrohydrodynamic replication of hexagonally ordered hole array nanopattern by adjusting the filling ratio, *f*. (a)–(f) SEM images (top) and corresponding simulated hole array pattern (bottom) showing the patterned morphology for different *f* (0.15–0.8). The scale bars represent 500 nm. (g) Relationship between *τ*_0_/*τ*_m_, *f*, and the corresponding replicated hole pattern fidelity. The filled and open circles indicate the morphologies obtained by experiment (a)–(h) and simulation (j) and (k), respectively. The light red region indicates the suitable range of parameters for obtaining perfect replication of hexagonally ordered hole nanopatterns. The insets show the simulated morphologies corresponding to the red filled circle (perfect replication) and red open circle (imperfect replication). The interfacial height of the simulated morphology is indicated by the color legends.

SEM images and the simulated morphology for *τ*_0_/*τ*_m_ = 0.11–470.96 (*f* = 0.15–0.8) are shown in the upper and lower rows of Fig. 4a–f, respectively, where the given parameters are valid for both. At low filling ratios (*f* = 0.15–0.35), nanoscale structures lacking periodic order formed over the film surface (Fig. 4a–c) since the growing pattern is governed by *λ*_m_ instead of *λ*_p_ due to the relatively low values of *τ*_0_/*τ*_m_. However, semi-periodic features occurred (Fig. 4d and e) when *τ*_0_/*τ*_m_ increased to 19.32 (*f* = 0.5). At a high filling ratio (>*f* = 0.65) and significantly increased *τ*_0_/*τ*_m_ (>96.94), the hole array features become clear. In particular, when *τ*_0_/*τ*_m_ was 470.96 (*f* = 0.8), which is of a higher order of magnitude than that in other cases (*f* < 0.8), the hole pattern was perfectly replicated, as shown in Fig. 4f. Fig. 4g summarizes the experimental results obtained *via* EHD-driven patterning with varying *f*. Unsuccessful/successful replication is indicated by the filled/open circles on the blue plot for *τ*_0_/*τ*_m_ as a function of *f*. As expected, the morphology approaches the perfectly replicated hole features with *τ*_0_/*τ*_m_ increasing with increasing *f*. To further extend the parametric regime for perfect replication in EHD-driven patterning, we simulated the relationship between *τ*_0_/*τ*_m_, *f*, and the corresponding hole feature morphology (red colored area). According to this parametric demarcation, even at *f* = 0.15, the hole pattern was successfully replicated by increasing *τ*_0_/*τ*_m_, which differs from the experimental results. In contrast, even if equal values of *f* were applied, only partial replication was confirmed from the simulation when *τ*_0_/*τ*_m_ was lower than that in the case of (f) (the blue filled circle in Fig. 4g). Consequently, it can be confirmed again that *τ*_0_/*τ*_m_ essentially determines perfect/imperfect replication.

Nanopattern replication employing EHD instability is dependent on the spatial distribution of the field strength, which is modulated by a topologically structured top electrode. Therefore, the structural properties of the master pattern should be rationally designed for facile and tunable replication such that they strongly affect the field strength over the entire film surface. Among the several parameters for determining the electric field (electrostatic pressure), the protrusion height (*h*_p_) is significant for the pattern replication mechanism; this is because the air gap (*d* − *h* − *h*_p_) reduced by *h*_p_ generates the highest field strength to enable the liquid thin film to be drawn under the protrusions. Considering 1/*τ*_m_, two different EHD instabilities in the pattern growth velocity are induced on the liquid thin film surface in the presence of the laterally varying electric field (note that homogeneous conductivity is assumed over the entire surface of the master stamp). On one hand, 1/*τ*_m_ is higher near the protrusions with protrusion height *h*_p_, *i.e.*, relatively fast pattern growth occurs in areas of the film above which there is a protruding master pattern feature with height *h*_p_; these areas are considered the feature domain of the film being patterned. On the other hand, 1/*τ*_n_ decreases with increasing distance from the protrusions; this corresponds to another reciprocal characteristic time in the absence of *h*_p_, *i.e.*, in areas of the film above which there is no protrusion in the top electrode, and this is known as the inter-feature domain of the film being patterned. To compare the different growth velocities under regions with and without protrusions, *i.e.*, in the feature and inter-feature domains, the ratio between 1/*τ*_m_ and 1/*τ*_n_ was taken as a new parameter, as follows:4
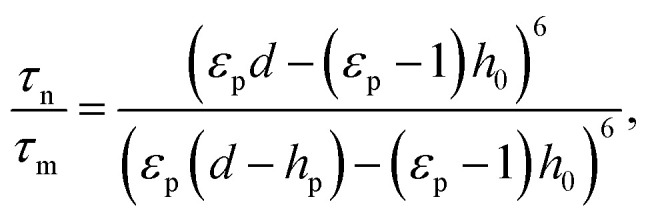


This parameter can be further expressed as5
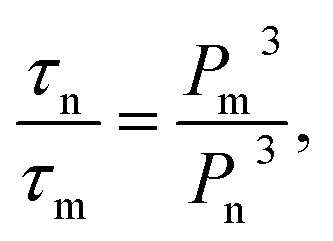
where *P*_m_ is the electrostatic pressure in the feature domain of the film, and *P*_n_ is the electrostatic pressure in the inter-feature domain during pattern replication. Intuitively, the more that *τ*_n_/*τ*_m_ increases, the more effectively the pattern is replicated in the feature domain; this is owing to the greater increase in *P*_m_ compared to that in *P*_n_. To confirm this, EHD-driven patterning was conducted using master patterns with different *h*_p_ (40, 120, and 300 nm) but the same lateral size (width and periodicity) and shape (line pattern with a linewidth of 300 nm and periodicity of 600 nm).

**Fig. 5:**
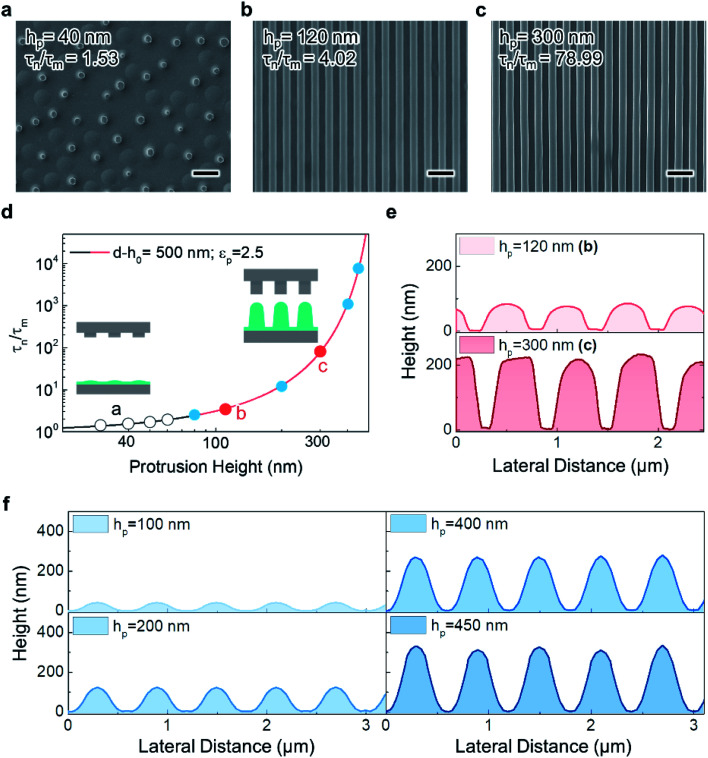
1/*τ*_m_-dependent replication of a line pattern by varying the protrusion height (*h*_p_) on the master stamp. (a)–(c) SEM images showing imperfect and perfect replication for *h*_p_ = 40, 120, and 300 nm, respectively (*τ*_n_/*τ*_m_ = 1.53, 4.02, and 78.99, respectively). The scale bars represent 10 μm in (a) and 1 μm in (b) and (c). (d) *τ*_n_/*τ*_m_ against *h*_p_ for *d* − *h*_p_ = 500 nm and *ε*_p_ = 2.5. The inset schematic drawings show cases of low and high protrusion height. Black open circles indicate imperfect replication or complete failure to replicate the given master pattern. Red and blue circles correspond to perfect replication in simulation and experiment, respectively. (e) Cross-sectional height profiles characterized by AFM. In (b) and (c), the pattern heights are approximately 80 and 225 nm, respectively. (f) Cross-sectional height profiles obtained from simulations for *h*_p_ = 100, 200, 400, and 450 nm.

The SEM images in Fig. 5a–c present the morphology obtained at different *τ*_n_/*τ*_m_ values of 1.53, 4.02, and 78.99, respectively, by adjusting *h*_p_ while maintaining the applied voltage (80 V). At *τ*_n_/*τ*_m_ = 1.72 (*h*_p_ = 50 nm), only microscale pillars were formed, and no line arrangement was observed; this is relatively similar to the case in which a homogeneous electric field is applied to a liquid thin film using a planar top electrode. This is clearer when considering the corresponding low *P*_m_/*P*_n_ (1.15), which is similar to that for the case in which a planar top electrode is employed, where *P*_m_/*P*_n_ = 1. In contrast, a perfectly replicated line array was observed at *τ*_n_/*τ*_m_ > 4.02 (*h*_p_ > 120 nm), as shown in Fig. 5b and c, where *P*_m_/*P*_n_ is > 1.59. The simulated morphology obtained by varying *τ*_n_/*τ*_m_ further confirms the influence of *τ*_n_/*τ*_m_ on the fidelity of the replicated features. Fig. 5d shows a plot of *τ*_n_/*τ*_m_ as a function of *h*_p_, in which the experimental and simulated results for unsuccessful (open black circles) and successful (closed circles) replications are represented. According to the simulated results, the patterns are replicated at *τ*_n_/*τ*_m_ > 2.4 (*P*_m_/*P*_n_ is > 1.34) when other parameters, such as the applied voltage (80 V) and dielectric constant (2.5), are fixed.

Importantly, in this experiment, the feature aspect ratio of the replicated pattern (*i.e.*, ratio of pattern feature height to width) also increased for higher *τ*_n_/*τ*_m_, achieved by using master pattern features with a higher aspect ratio (*h*_p_). For *τ*_n_/*τ*_m_ = 4.02 and 78.99, despite the similar lateral features of the replicated patterns, the cross-sectional topologies, as measured using atomic force microscopy (AFM), are considerably different. For *τ*_n_/*τ*_m_ = 4.02 (*h*_p_ = 120 nm), the replicated line array pattern exhibits a low feature height of approximately 80 nm (aspect ratio ≈ 0.27), as shown in Fig. 5e (upper). However, for *τ*_n_/*τ*_m_ = 78.99 (*h*_p_ = 300 nm), the feature height is significantly higher (225 nm, aspect ratio: 0.75) (Fig. 5e (lower)). Given that *P*_m_/*P*_n_ is higher (4.29) for an increased *h*_p_ (300 nm), the increased strength of the electrostatic pressure induced by the increased *h*_p_ more effectively draws the film liquid to replicate the protruding features of the master stamp. The simulated results indicated by the blue circles in Fig. 5d further support this explanation. Fig. 5f shows the replicated pattern feature height trend corresponding to an increasing master pattern feature height.

Conventionally, the features replicated by EHD-driven patterning exhibit a low aspect ratio of <1, especially for nanoscale features, when the dielectric constant of the patterned film is not modified;^46^ this restricts the diversity of potential applications of patterns obtained by EHD-driven patterning, which in turn affects the applicability of EHD-driven patterning as a fabrication tool. However, this issue may be overcome by carefully adjusting *h*_p_, as shown in the simulated data in Fig. 5f (aspect ratio: 1.1 at *h*_p_ = 450 nm). Therefore, proper care should be taken when designing the structure of the master stamp such that reliable replication of nanopatterns with increased height and aspect ratio can be facilitated.

**Fig. 6:**
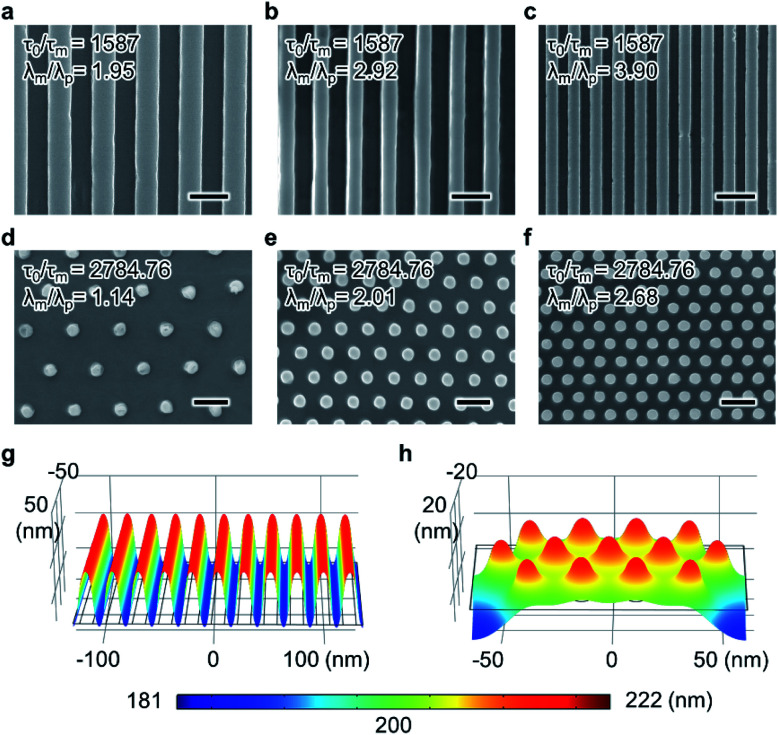
*λ*_m_-independent replication of line and hexagonally ordered pillar patterns by varying the master pattern periodicity (*λ*_p_). (a)–(c) SEM images of three replicated line patterns, each with a different linewidth, for *τ*_n_/*τ*_m_ = 1587. The scale bars represent 500 nm. (d)–(f) SEM images of three pillar patterns, each with a different density, at *τ*_n_/*τ*_m_ = 2784.76. The scale bars represent 500 nm. (g) and (h) Simulated 3D surface topology in 10 nm-sized nanopattern replication through the rapid EHD-driven patterning route. Successful replication of line array with 10 nm linewidth (g) and hexagonally ordered pillar pattern with 10 nm diameter (periodicity of 20 and 27 nm, respectively) (h) for *τ*_n_/*τ*_m_ = 126 966 in both cases. The interfacial height is indicated by the color legends.

To further confirm that the influence of *λ*_m_/*λ*_p_ on perfect replication is reduced by increasing 1/*τ*_m_, master patterns of different sizes and densities were replicated using EHD-driven patterning while maintaining a high *τ*_0_/*τ*_m_ (1587 and 2,785, respectively). The influence on feature size was examined by replicating three nanoscale line arrays (linewidths of 300, 200, and 150 nm). All parameters, except the geometry of the master patterns, were fixed at *τ*_0_/*τ*_m_ = 1587, although each case corresponded to a different *λ*_m_/*λ*_p_ (1.95, 2.92, and 3.90, respectively) because of the different periodicities of the master patterns (*λ*_p_ = 600, 400, and 300 nm, respectively). Despite the different *λ*_m_/*λ*_p_ values for each case, line array patterns with different linewidths were replicated using the same parametric conditions corresponding to *τ*_0_/*τ*_m_ (Fig. 6a–c). To inspect the effect of pattern density, three types of hexagonal pillar nanopatterns were used, each featuring the same pillar diameter of 150 nm but with different feature-to-feature spacings (700, 400, and 300 nm). Again, despite the different *λ*_m_/*λ*_p_ values for each case (1.14, 2.01, and 2.68, respectively), EHD-driven patterning was performed with the same *τ*_0_/*τ*_m_ (2784.76). The SEM images of the resulting replicated pattern morphology confirm that the three different master patterns were successfully replicated, as shown in Fig. 6d–f. Therefore, increasing 1/*τ*_m_ in EHD-driven patterning not only minimizes the importance of *λ*_m_–*λ*_p_ matching in achieving perfect replication but also implies enhanced scalability at the nanoscale.

Although there is no theoretical achievable resolution limit in EHD-driven patterning, the smallest reported nanopattern feature size is 50 nm,^22^ and no parametric guide for replicating nanoscale features below the 50 nm length scale has been proposed. The difficulty in EHD-driven patterning stems from the increased Laplace pressure that occurs for smaller patterns, which dampens the thin film surface undulations. Increasing 1/*τ*_m_ assists in replicating small features because the air gap, which is reduced by the rapidly amplified undulation at an early stage, increases the strength of the electrostatic pressure, thereby reducing the influence of the Laplace pressure.

Owing to the lack of a suitable master stamp, conducting rapid EHD-driven patterning to experimentally replicate nanopatterns with small features at the 10 nm length scale was not possible. Nevertheless, the morphology of 10 nm-scale replicated features was simulated by adjusting parameters to increase 1/*τ*_m_. Note that the main parameters with regard to 1/*τ*_m_, such as the applied voltage and air gap, were limited to the allowed parameters in our EHD-driven patterning system, specifically *U* < 120 V and 50 nm < *d* − *h*_0_ < *d*. As shown in Fig. 6g and h, according to the simulation, two types of nanopatterns, a line array with a 10 nm linewidth and 20 nm periodicity and a hexagonal pillar array with a 10 nm pillar diameter and 27 nm periodicity, are predicted to be replicated for *τ*_0_/*τ*_m_ = 126 966. Considering these simulation results coupled with the experimental results in Fig. 6a–f, rapid EHD-driven patterning based on increasing *τ*_0_/*τ*_m_ exhibits promising scalability for small and dense nanoscale features down to the 10 nm scale.

## Conclusions

In this study, the parametric influence of EHD-driven patterning on nanopattern replication was explored with a focus on increasing 1/*τ*_m_ for rapid pattern growth. In conventional EHD-driven patterning, reliable replication depends on *λ*_m_–*λ*_p_ matching, which is technically difficult to achieve; this in turn hinders the potential applications of EHD-driven patterning. In this study, diverse nanopatterns were perfectly replicated with increased 1/*τ*_m_, even under conditions unfavorable for conventional EHD-driven patterning (*e.g.*, *λ*_m_/*λ*_p_ > 1); this is because the high pattern growth velocity contributed to preserving the periodic features that appeared in the early stages of pattern growth. Increasing 1/*τ*_m_, instead of matching *λ*_m_ to a given *λ*_p_ (or *vice versa*), resulted in perfect replication of nanopatterns without sacrificing pattern quality. Simulations in which several parameters were varied with respect to 1/*τ*_m_ confirmed the experimental results and were used to delimit the parameter domains that yield perfect and imperfect replication. The resultant predictive diagram is expected to provide technical guidance for pattern transfer using EHD-driven patterning. Rapid EHD-driven patterning not only facilitates nanopattern replication across a wide range of parameters, but also enhances the scalability and geometric controllability of the replicated features. Furthermore, the feasibility of replicating nanopatterns with 10 nm features using rapid EHD-driven patterning has been confirmed for the first time. Therefore, nanoscale EHD-driven patterning with a high 1/*τ*_m_ may present an excellent opportunity for fundamental studies on electrically destabilized liquid thin films as well as for industrial applications of large-area uniform patterning at the sub-10 nm level.

## Conflicts of interest

The authors declare no competing financial interest.

## Supplementary Material

RA-011-D1RA01728D-s001
