# Supplementary material for: Parametric scheme for rapid nanopattern replication via electrohydrodynamic instability
Source: RSC Adv. 2021 May 19;11(30):18152–61. doi: 10.1039/d1ra01728d (PMC9033445; doi:10.1039/d1ra01728d)
Supplement: RA-011-D1RA01728D-s001 [file RA-011-D1RA01728D-s001.pdf]

Electronic Supplementary Material (ESI) for RSC Advances.
This journal is © The Royal Society of Chemistry 2021

Electronic Supplementary Information

Parametric Scheme for Rapid Nanopattern Replication via Electrohydrodynamic Instability

Jaeseok Hwang[1,#], Hyunje Park[2,#], Jaejong Lee[3,*] and Dae Joon Kang[2,*]

[1]Department of Energy Science, Sungkyunkwan University, 2066, Seobu-ro, Jangan-gu, Suwon, Gyeonggi-do 16419, Republic of Korea

[2]Department of Physics, Sungkyunkwan University, 2066, Seobu-ro, Jangan-gu, Suwon, Gyeonggi-do 16419, Republic of Korea

[3]Korea Institute of Machinery and Materials (KIMM), 156 Gajeongbuk-ro, Yuseong-gu, Daejeon 34103, Republic of Korea

Fax: +82-(31)-290-5906 E-mail addresses: *djkang@skku.edu* ; *jjlee@kimm.re.kr*

[*]Corresponding author.

[#]Both authors contributed equally to this work.

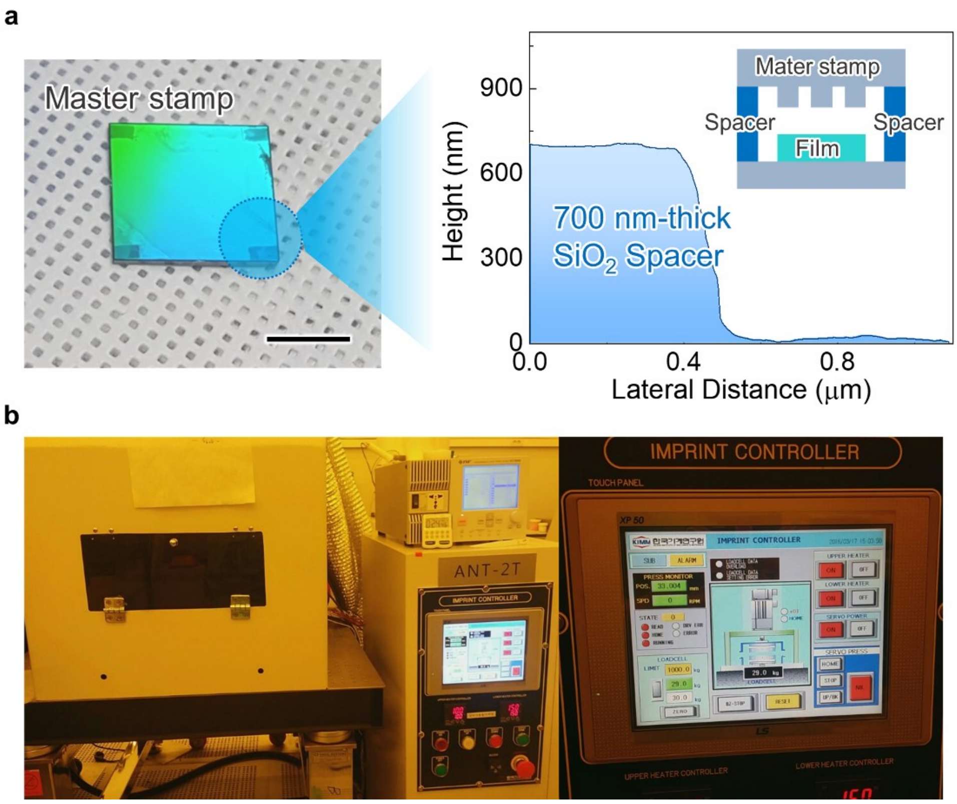

Fig. S1. (a) Dielectric spacers placed on each four corners of the master stamp are employed to support the wedge geometry in capacitor-like assembly. The electrode gap of 400 nm was maintained by employing 700 nm-thick spacer layer and the protrusion height of 300 nm (i.e., 700 – 300 = 400 nm). The thickness of spacer layer (i.e., electrode gap) was measured by AFM. Scale bar represents 1 cm. (b) To maintain gap uniformity during pattern replication, a commercially available nano-imprinter was employed with a mechanical pressure of 50 kPa.

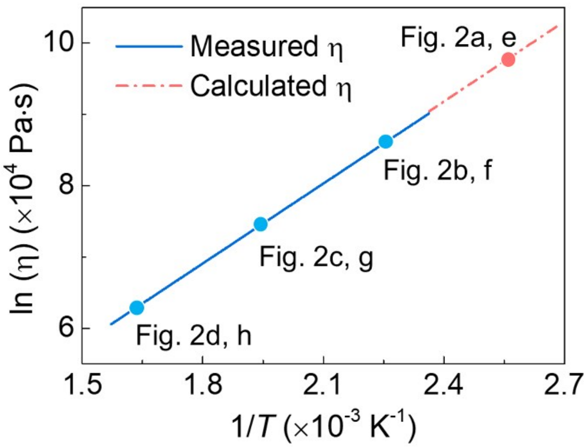

Fig. S2. The viscosity values of the liquefied PS film were measured by using the Rheometer system (ARES-G2; shear rates of 0.01 – 0.1 s^{-1} at 0.1 °C/s). The measured viscosity values (blue solid line) are in excellent agreement with our calculated ones (red dash-single dotted line). It should be also noted that, because of the limited fluidity of PS below 150 °C, the calculated value of η at 120 °C was used instead for the sake of completeness.

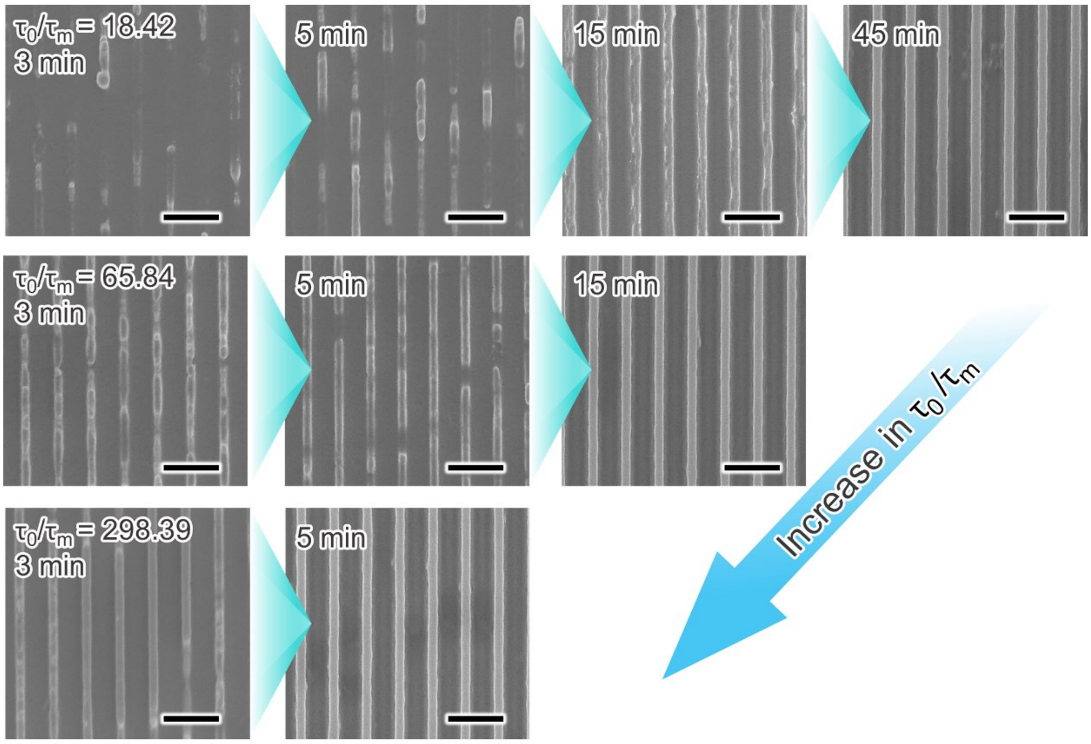

Fig. S3. The effect of τ_0/τ_m on pattern evolution: a nano-sized line array having 200 nm linewidth and 550 nm periodicity was successfully replicated in 45 min at τ_0/τ_m = 18.42. Further increase of τ_0/τ_m up to 298.39, the faithful replication of the line array was completed in 5 min. Scale bar, 1 μm.
